# Responsible use of negative research outcomes—accelerating the discovery and development of new antibiotics

**DOI:** 10.1038/s41429-021-00439-w

**Published:** 2021-07-16

**Authors:** Helen Yu

**Affiliations:** Centre for Advanced Studies in Biomedical Innovation Law, Faculty of Law, University of Copenhagen, Copenhagen, Denmark

**Keywords:** Publishing, Drug development, Health care

## Abstract

Failure to share and make use of existing knowledge, particularly negative research outcomes, has been recognized as one of the key sources of waste and inefficiency in the drug discovery and development process. In the field of antibiotic research, providing a platform where negative outcomes could be shared to prevent the vicious cycle of duplicating costly studies that produce the same negative results would greatly de-risk and accelerate the development of new antibiotics. Providing a legally supported framework that recognizes negative outcomes as intellectual contributions, which can subsequently be translated into a revenue-sharing model, may lead to more openness and value creation in support of a sustainable and responsible transformation of research into socially and economically beneficial innovations.

## Introduction

In a report by the European Patent Office, an estimated USD$20 billion are spent every year to develop innovations and technologies that have already been developed elsewhere [1], highlighting the exorbitant cost of duplication arising from the lack of sharing existing knowledge. In the context of drug discovery and development, failures have been identified as a key contributor to the significant costs associated bringing new drugs to market [2]. Equally discouraging is the finding that over 85% of research funds, equivalent to USD$100 billion per year globally, are wasted per year due in large part to selective non-publication and poor reporting of information that should otherwise be publicly available, leading to ineffective uptake and application of research and findings [3]. Despite these alarming numbers, the development of open innovation efforts [4], accompanied by research and innovation policies in support of collaborative open access to research and sharing of available knowledge, there is a general acceptance by stakeholders of the innovation ecosystem of efforts directed at reducing research waste and accelerating innovation [5], particularly in the discovery and development of new antibiotics.

### Negative research outcomes

Research failures in the context of drug discovery and development are understood to mean a negative outcome or failed clinical trial where the results do not demonstrate positive safety and/or efficacy of an investigational drug [2]. Negative outcomes could also arise from flawed designs or poorly executed clinical trials where the results, even if positive, are deemed to be a failure. Regardless of the reason for failure, the competitive nature of the pharmaceutical industry is such that the voluntary sharing of information, even negative outcomes, may not be particularly attractive or forthcoming if withholding means delaying competitors by not helping them avoid incur the time and cost of making known mistakes [6]. Although, there is some evidence of an increase in willingness to publish and share negative outcomes, failure to share and mobilize existing knowledge has been recognized by the research community as one of the key sources of waste in the research process which could be used to prevent the vicious cycle of other researchers wasting time and public funds to duplicate studies that produce the same negative results [7]. For example, one study found strong evidence that information on adverse events related to medical treatments remain unpublished in journals, leaving health professionals, policy makers, and patients unable to make informed decisions [8]. Another study looking at investigational drugs entering late-stage clinical testing found that more than half of the negative outcomes attributed to inadequate drug efficacy or safety remained unpublished [9]. Without this knowledge to inform research and clinical development, the transformation of science into new innovations may be hindered without the benefit of insights from negative outcomes to divert investigational attention to more productive targets [10]. However, because negative outcomes are considered ‘useless’ data to the party owning it but highly valuable to the competition, if the value of negative outcomes can be captured, the sharing of research failures may be perceived as a less competitively threatening if the timely sharing of unsuccessful research results can be incentivized [6].

### Responsible knowledge commons platform for sharing negative outcomes

Existing initiatives that encourage open sharing in antibiotic discovery and development (see for example Combating Antibiotic-Resistant Bacteria https://carb-x.org/ and Shared Platform for Antibiotic Research and Knowledge https://www.pewtrusts.org/en/research-and-analysis/articles/2018/09/21/the-shared-platform-for-antibiotic-research-and-knowledge) have yet to demonstrate wide success. The sustainability of such initiatives has often been questioned because of the long timelines in drug discovery and development, as well as the challenge of establishing a balance between public and private interests, particularly with respect to the management of intellectual property (IP) [11]. Because intellectual efforts that contribute to the creation of innovations, particularly data, may not fall within the scope of IP protection as traditionally defined, innovators and researchers may be more likely to safeguard their personal interests at the expense of sharing knowledge if there is no incentive to contribute or if there is inadequate protection of their proprietary information [6]. There needs to be a legally supported knowledge sharing platform that recognizes the contributions of data or proprietary information to incentivize the sharing of negative outcomes to accelerate the discovery and development of new antibiotics.

Trade secret protects against the unlawful acquisition, use, and disclosure of information and know-how that has value, such as data that provides a competitive advantage or allows its owner to generate profit. (See European Parliament. Directive (EU) 2016/943 on the protection of undisclosed know-how and business information (trade secrets) against their unlawful acquisition, use and disclosure; see also Uniform Law Commission, United States Uniform Trade Secret Act, 14 ULA (1985) §1.4). Rarely discussed is the legal protection of data and knowledge arising from negative outcomes and the vital role such protection plays in the value of sharing failures because of its ability to reduce wasteful duplication efforts. Negative trade secrets essentially include information on why an innovation does not work—what went wrong and why. Negative outcomes, particularly in the field of drug discovery and development, are therefore good candidates for trade secret protection because they are: (a) considered highly valuable to the competition because it can translate into significant savings by eliminating known and costly missteps; and (b) they are complex and not likely to be discovered independently without significant investment in time and resources [6]. For example, the transparency rules of the EU Clinical Trial Regulation specifically recognize that clinical trial data may contain “commercially confidential information” and may be subject to redaction [12]. In the US where similar transparency rules on clinical trials exist, the FDA has recently taken the active step of enforcing the legal requirement to submit clinical trial results to ensure compliance with reporting obligations [13], while recognizing that trade secret and/or confidential commercial information may be redacted. As such, negative clinical trial outcomes and adverse events may benefit from negative trade secret protection and the sharing of such information could be incentivized through a knowledge commons type platform.

Knowledge commons are a recognized means to pool and share existing knowledge under a common property framework with the objective of keeping access to information open to facilitate the creation of new innovations [14]. In the context of drug discovery and development, knowledge commons have been criticized as having limited utility because it is unlikely that genuinely valuable information and knowledge will be shared given the cost of innovation and the competitive advantage of keeping knowledge secret [15]. However, by combining blockchain technology and trade secret protection with knowledge commons to manage the sharing of negative outcomes, a legally supported knowledge sharing platform that encourages speed to market competition based on who can innovate from known failures can be created [6].

Applied to antibiotics discovery and development, finding a way to leverage negative outcomes as a protected negative trade secret to create positive value through coopetition, may be a viable incentive model to proactively encourage and optimize the use of existing high-quality data. This can be achieved through a membership-based “research failures knowledge commons” platform for sharing negative trade secrets where users who successfully innovate through avoiding negative outcomes would be allowed to retain and preserve IP rights in their successes, but research failures (as a proprietary negative trade secret) must be contributed back as a condition of access to the platform [6]. This “research failures knowledge commons” will ensure that members who want access to applicable negative outcomes must also contribute their associated research failures. For those willing to share, under obligations of confidentiality, trade secret protection promotes participation in the knowledge commons by: (a) providing assurance that contributions will be properly attributed and credited with the help of blockchain technology; and (b) providing access to relevant research failures to learn from and navigate around known hurdles. The cumulative effect of intellectual contributions and the associated public good of sharing negative knowledge (as a protected right) for the benefit of accelerating research and innovation will keep the pathways to discovery much more open while preserving exclusionary rights to the discovery of successes.

## Discussion

One of the core objectives of RRI policy is to maximize the value of publicly funded research so it may be returned to the benefit of society [6]. To ensure that negative outcomes arising particularly from publicly funded efforts into the discovery and development of new antibiotics are made available for the common public good, RRI principles should be interpreted and applied by funding agencies to demand mandatory reporting of negative research results as a condition of receiving public funding. Funders can then make such reports publicly available should journals and academic publishers decline to publish negative outcomes. Failure to share and mobilize existing knowledge has been recognized by the research community as one of the key sources of waste in the research process [7]. Fortunately, there is some evidence of an increase in willingness to publish and share negative outcomes [9], which could be used to prevent the vicious cycle of other researchers wasting time and public funds to duplicate studies that produce the same negative results. For example, in the field of medicinal chemistry, failed experiments and reactions are often published [16], setting an example for other scientific fields to follow. However, the long tradition of publication bias still means positive results are selectively being reported [17]. In part, the problem stems from researchers not wanting to invest the time in preparing articles reporting on negative outcomes in fear that publishers will reject them in favor of works with successful and positive results, which are cited more often [18, 19]. The consequence of not publishing negative outcomes combined with the potential positive bias of published results is the potential to misinform the scientific community [17]. Another study looking at investigational drugs entering late-stage clinical testing found that more than half of the negative outcomes attributed to inadequate drug efficacy or safety remained unpublished [9]. Without this knowledge to inform drug research and development, the transformation of science into new innovations may be hindered without the benefit of insights from negative outcomes to divert investigational attention to more productive targets.

In addition to avoiding waste and duplication, there is also an implied obligation that publicly funded research conducted for the benefit of the public should be published or reported as part of the responsibility and accountability of researchers to society [14]. The Netflix-style subscription model to incentivize antibiotics research and development has been discussed as a way to de-link the traditional volume-based profit model from the ability of pharma to recoup sunk costs in novel antibiotic research and development [11]. By paying companies upfront for a defined volume of drugs to be stored in reserve for future use instead of being paid for volumes actually prescribed, investment in the discovery of new antibiotics can be encouraged. Although this model addresses the financial concerns related to the commercial viability of making new antibiotics available, it fails to address and incentivize the sharing of existing knowledge to accelerate the innovation process itself. By incentivizing the sharing of negative research outcomes by offering the ability to recuperate part of the sunk costs associated with negative outcomes, even if the successful innovation comes from a third party, the innovation process can be de-risked.

## Conclusion

A knowledge sharing platform that proactively tracks contributions to an innovation that can subsequently translate into a revenue-sharing model may lead to more openness and overall acceleration of antibiotic discovery and development.
